# Therapeutic Effect and Optimal Electrode Placement of Transcutaneous Neuromuscular Electrical Stimulation in Patients with Post-Stroke Dysphagia: A Systematic Review and Meta-Analysis of Randomized Controlled Trials

**DOI:** 10.3390/life12060875

**Published:** 2022-06-10

**Authors:** Thanh-Nhan Doan, Wen-Chao Ho, Liang-Hui Wang, Fei-Chun Chang, Trang Thi Quynh Tran, Li-Wei Chou

**Affiliations:** 1 Department of Public Health, China Medical University, Taichung 406040, Taiwan; u110050102@cmu.edu.tw (T.-N.D.); wcho@mail.cmu.edu.tw (W.-C.H.); 2 Department of Rehabilitation, Quang Nam Northern Mountainous Region General Hospital, Quang Nam 560000, Vietnam; 3 Department of Speech Language Pathology and Auditory, HungKuang University, Taichung 433304, Taiwan; wlhui0815@sunrise.hk.edu.tw; 4 Ph.D. Program for Aging, China Medical University, Taichung 404332, Taiwan; d22114@mail.cmuh.org.tw; 5 Department of Physical Medicine and Rehabilitation, China Medical University Hospital, Taichung 404332, Taiwan; 6 Faculty of Rehabilitation, University of Medicine and Pharmacy, Hue University, Thành phố Huế 530000, Vietnam; ttqtrang@huemed-univ.edu.vn; 7 Department of Physical Therapy and Graduate Institute of Rehabilitation Science, China Medical University, Taichung 406040, Taiwan; 8 Department of Physical Medicine and Rehabilitation, Asia University Hospital, Asia University, Taichung 413505, Taiwan

**Keywords:** neuromuscular electrical stimulation, dysphagia, stroke, electrode placement, swallowing therapy

## Abstract

Background: To date, there is no conclusive evidence that transcutaneous neuromuscular electrical stimulation (TNMES) benefits patients with post-stroke dysphagia (PSD). In addition, the optimal TNMES electrode placement has not been well-established. This systematic review and meta-analysis were conducted to investigate these two research gaps. Methods: Five major databases were systematically searched for randomized controlled trials (RCTs) through January 2022. Effect sizes were computed using Hedges’ g statistic, which were then entered into the random-effects model to obtain pooled effect estimates. Results: Twenty-four RCTs met the eligibility criteria. On the improvement of swallowing function, TNMES alone was not superior to conventional swallowing therapies (CSTs); combined therapy of TNMES and CSTs significantly surpassed CSTs alone (standardized mean difference (SMD) = 0.91, 95% confidence interval (95% CI): 0.68 to 1.14, *p* < 0.0001; I^2^ = 63%). Moreover, significant pooled effect sizes were observed in subgroups with horizontal electrode placement above the hyoid bone (SMD = 0.94, 95% CI: 0.72 to 1.16; I^2^ = 0%) and horizontal electrode placement just above and below the hyoid bone (SMD = 0.87, 95% CI: 0.59 to 1.14; I^2^ = 0%). The largest pooled effect size was observed in the subgroup that individualized electrode placement according to dysphagia evaluation (SMD = 1.65, 95% CI: 0.38 to 2.91; I^2^ = 90%). Conclusion: TNMES should be used in combination with CSTs for PSD. Horizontal electrode placement should target suprahyoid muscles or both suprahyoid and thyrohyoid muscles.

## 1. Introduction

Stroke, also referred to as a cerebrovascular accident, is a common and debilitating disease with a high prevalence of disability and fatality [1]. Post-stroke dysphagia (PSD), which is defined as difficulty swallowing after stroke, is one of the most common complications, affecting nearly half of stroke survivors [2]. PSD is associated with various devastating consequences, including malnutrition, water–electrolyte imbalance, aspiration, pneumonia, prolonged length of hospital stay, psychological distress, reduced quality of life, and increased risk of mortality [2,3]. Over the past two decades, the area of neuroplasticity has become increasingly popular, especially the mechanism of swallowing function with the immense complexity of neural circuits is supposed to respond considerably to rehabilitative treatment [4,5]. While conventional swallowing therapies (CSTs) such as compensatory strategies, texture-modified diet, postural adjustment, or swallow maneuvers are widely administered for patients with PSD [6], the effect of combination therapy of CSTs and transcutaneous neuromuscular electrical stimulation (TNMES) remains debatable. A previous meta-analysis searched PubMed and Scopus libraries up to 31 December 2014 for both randomized controlled trials (RCTs) and quasi-experimental studies administering TNMES for patients with PSD; a significant effect size favoring swallowing therapy with TNMES over swallowing therapy without TNMES was found [7]. Nevertheless, there was significant heterogeneity among eight included studies (I^2^ = 85%) [7]. A recent systematic review and meta-analysis searched through August 2019 drew an ambiguous conclusion on the effectiveness of TNMES in dysphagia caused by various etiologies [8]. In accordance with the National Institute for Health and Care Excellence (NICE), evidence of TNMES for patients with PSD is still limited in quality and quantity [9]. TNMES carries electrical impulses to stimulate muscular contraction, improving or restoring the function of stimulated muscles [10]. The supra and infrahyoid muscles have been widely described as contributing to swallowing function [11]. Depending upon electrode placements in the submental or laryngeal regions, TNMES activates suprahyoid muscles, which cause the hyoid bone to move anterosuperiorly, whereas stimulating infrahyoid muscles depresses the hyoid bone and the hyolaryngeal complex. The thyrohyoid muscle, one of the four infrahyoid muscles, raises the larynx when the suprahyoid muscles stabilize the hyoid bone. Furthermore, by moving the hyoid bone anterosuperiorly, the thyrohyoid and suprahyoid muscles aid in the opening of the inferior pharyngeal sphincter [12,13]. Different studies applied varying electrode placements that might produce significant heterogeneity among them. Therefore, we conducted this systematic review and meta-analysis to verify the efficacy and identify the optimal electrode placement of TNMES in patients with post-stroke dysphagia.

## 2. Materials and Methods

This systematic review and meta-analysis was registered on the International Prospective Register of Systematic Reviews (PROSPERO identifier: CRD42022299184) and reported in accordance with the Preferred Reporting Items for Systematic Reviews and Meta-Analyses (PRISMA) guidelines [14]. The first author conducted the search strategies. Full-text analyses, study selection, data extraction, and methodological quality assessment were carried out by two authors and verified by the other authors. Consensuses were reached through group discussion when any discrepancies arose.

### 2.1. Eligibility Criteria

A systematic search was conducted to examine all potential RCTs published in English, identifying the effect of TNMES in patients with post-stroke dysphagia. Observational studies, nonhuman studies, quasi-experiments, abstracts, or conference proceedings with no full-text were excluded.

Types of population: We included trials with patients who experienced stroke irrespective of stages with dysphagia clinically diagnosed. We omitted studies involving individuals with diagnoses other than stroke or studies that used self-report questionnaires to determine dysphagia.

Types of interventions and comparators: We looked for RCTs that compared TNMES with CSTs, compared combined intervention of TNMES and CSTs with CSTs alone, or compared different electrode placements of TNMES. We excluded studies that combined TNMES with other physical modalities such as transcranial direct current stimulation, repetitive transcranial magnetic stimulation, functional magnetic neuromuscular stimulation, or studies that used electrical stimulation other than transcutaneous neuromuscular electrical stimulation such as pharyngeal electrical stimulation.

Types of outcomes: The primary outcome of this systematic review was swallowing function assessed by instrumental swallowing assessments such as videofluoroscopic swallow study (VFSS) or fiberoptic endoscopic evaluation of swallowing (FEES), bedside swallowing assessments such as Standardized Swallowing Assessment (SSA), Volume-Viscosity Swallowing Test (V-VST), Gugging Swallow Screening (GUSS), Mann Assessment of Swallowing Ability (MASA), Water Swallow Test (WST), and Functional Oral Intake Scale (FOIS). Secondary outcomes could be dysphagia-related health outcomes such as the Swallowing-Related Quality of Life Questionnaire (SWALQOL).

### 2.2. Search Strategy and Screening Process

A systematic search was conducted from inception until January 2022 of Pubmed, Excerpta Medica dataBASE (EMBASE), Web of Science, Cumulative Index of Nursing Allied Health Literature (CINAHL), and Cochrane libraries. The specific search strings used on each library are detailed in Table S1. In addition, we considered reference lists of relevant articles to obtain more potential papers. Initially, duplicates were automatically excluded by EndNote X9. Irrelevant research was subsequently ruled out by title and abstract examination. Two reviewers separately read full texts of potential articles to collect studies that met the eligibility criteria. All uncertainties were resolved by group discussion.

### 2.3. Assessment of the Risk of Bias

The risk of bias of the selected studies was assessed independently by two authors using the Physiotherapy Evidence Database (PEDro) scale. On a scale of 0–10, this scale rates the methodological quality of RCTs. High, moderate, and low methodological quality were assigned to studies with scores of at least six, four to five, and less than four, respectively [15]. Any discrepancies arising between the two raters were resolved through group discussion.

### 2.4. Data Extraction

We extracted the study characteristics comprising the first author’s surname, year of publication, country, sample size, duration since stroke onset, intervention protocol of TNMES including pulse duration, frequency, intensity, number of sessions, duration of a session, intervention duration, and electrode placements. In order to conduct meta-analyses, we extracted the primary outcomes with mean and standard deviation of pre- and post-intervention in the intervention group and control group. When the mean and standard deviation were not presented directly, we calculated mean and standard deviation from the available raw data or median and quartiles using the formulation of Wan et al., 2014 [16]. When more than one measure was utilized to assess swallowing function in a study, the primary outcomes were chosen in descending order of priority as follows: instrumental measures, bedside swallowing assessments, and FOIS.

### 2.5. Statistical Analysis

We used R version 3.6.2 (R Foundation for Statistical Computing, Vienna, Austria) to analyze the data. The effect size and 95% confidence interval were calculated by means and standard deviations using Hedges’ g statistic. The magnitude of an effect was defined as small, moderate, and large, corresponding to the value of 0.2, 0.5, and 0.8, respectively [17]. The random-effect model was constructed to pool the effect sizes, considering that clinical heterogeneity among the included studies was reasonable. The I^2^ and Q statistic were used to estimate the heterogeneity among included studies, I^2^ values of 25%, 50%, and 75% correspond to low, moderate, and high levels of heterogeneity [18]. If there was significant heterogeneity among included studies, we conducted subgroup analyses to explore the sources of heterogeneity, and narrative analysis was adopted in case considerable heterogeneity remained. We created forest plots to illustrate individual component effect sizes and confidence intervals, pooled effect size, and heterogeneity. The funnel plot and Egger’s test were carried out to assess publication bias [19]. The statistical significance was defined as a threshold of *p* < 0.05.

## 3. Results

**Figure 1:**
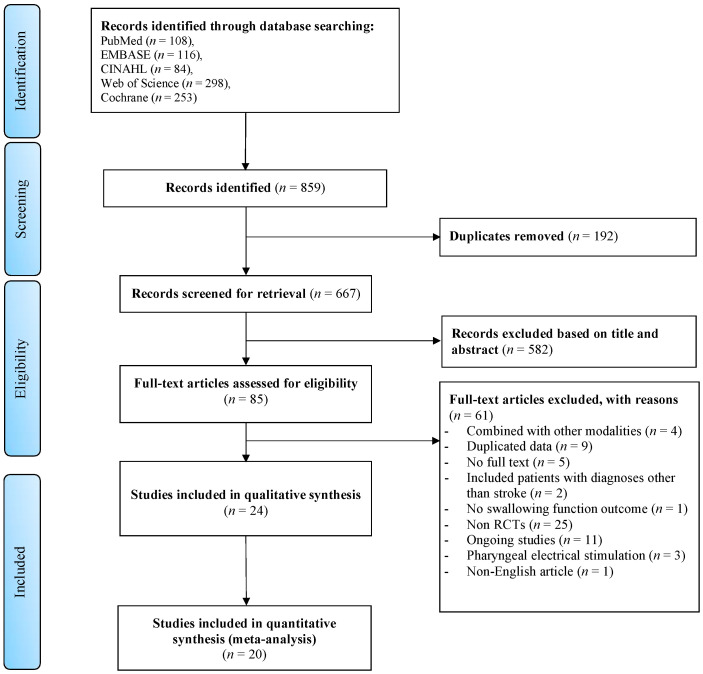
PRISMA flow diagram of the selection process for the systematic review and meta-analysis.

The systematic search through five electronic databases yielded a total of 859 articles, of which 192 studies were duplicated. After reviewing the titles and abstracts, we eliminated 582 studies that were not relevant. Of the remaining 85 studies that went through full-text analysis, 61 were further discarded for reasons: TNMES combined with other modalities (*n* = 4), duplicated data (*n* = 9), no full text available (*n* = 5), patients with diagnoses other than stroke (*n* = 2), no swallowing function outcome (*n* = 1), non RCTs (*n* = 25), ongoing studies (*n* = 11), pharyngeal electrical stimulation (*n* = 3), and non-English article (*n* = 1). Other than the identified studies, a grey literature search yielded no additional articles. Finally, 24 studies matched the criteria for inclusion in this systematic review, with 20 studies having sufficient data for meta-analysis. Figure 1 depicts the selection procedure.

### 3.1. Risk of Bias Assessment

The PEDro score found that 19 studies were categorized as good (6–8 scores) and 5 as moderate (5 scores). The 24 studies have a mean methodological quality score of 6.6 (SD ± 1.1, range: 5–8) on the PEDro scale. The risk of bias of the included studies is detailed in Table S2.

### 3.2. Characteristics of Studies

**Figure 2:**
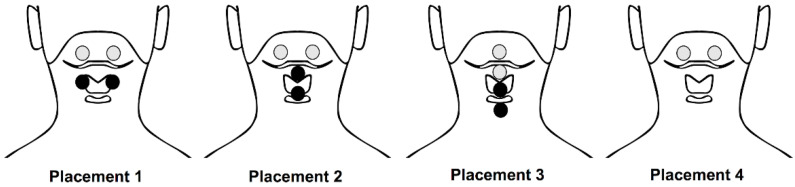
Electrode placements were applied most frequently among the included studies. Placement 1: horizontal electrode placement with one pair just above the hyoid bone and the other pair below the hyoid bone at the level of the thyroid notch over the thyrohyoid muscle. Placement 2: one pair of electrodes horizontally aligned above the hyoid bone and the other pair vertically placed below the hyoid bone. Placement 3: two pairs of electrodes vertically placed along the midline with one electrode just above the hyoid bone and the other three electrodes below the hyoid bone. Placement 4: one pair of electrodes horizontally aligned in the submental region.

This systematic review included 24 RCTs, including 1 study comparing TNMES to CSTs [20], 2 studies comparing TNMES combined CSTs to TNMES alone and CSTs alone [21,22], 18 studies comparing TNMES combined CSTs to CSTs alone [23,24,25,26,27,28,29,30,31,32,33,34,35,36,37,38,39,40], and 3 studies comparing the effect of TNMES with different electrode placements [41,42,43]. Regarding TNMES parameters, the majority of included studies set the frequency of 80 Hz, the most common pulse duration was 700 microseconds, with the intensity at the motor threshold that patients could tolerate. Despite some minor alterations, the frequency, pulse duration, and intensity administered in all included studies were suitable for reaching the motor contraction threshold. TNMES was administered in 20, 30, 40, or 60 min per day, with 30 and 60 min being administered more frequently. The number of intervention sessions ranged from 10 to 40, with the most typical frequency being five times per week across three or four weeks. Concerning electrode placements, Figure 2 illustrates four placements applied most frequently among the included studies. Six trials [20,24,27,28,33,40] horizontally aligned two pairs of electrodes, including one pair just above the hyoid bone and the other pair below the hyoid bone at the level of the thyroid notch over the thyrohyoid muscle (Placement 1); two trials [25,34] horizontally aligned one pair of electrodes above the hyoid bone and vertically placed the other pair below the hyoid bone (Placement 2); three trials [21,23,36] vertically placed two pairs of electrodes along the midline with one electrode just above the hyoid bone and the other three electrodes below the hyoid bone (Placement 3); seven trials [26,29,32,35,37,38,39] horizontally aligned one pair of electrodes in the submental region (Placement 4); one trial [30] placed two pairs of electrodes targeting infrahyoid muscles as resistance training; and the last two trials [22,31] individualized electrode placement for each patient based on dysphagia assessment results. The characteristics of the included studies are detailed in Table 1.

### 3.3. Meta-Analysis

#### 3.3.1. Therapeutic Effect of Combined TNMES and CSTs on Swallowing Function

**Figure 3:**
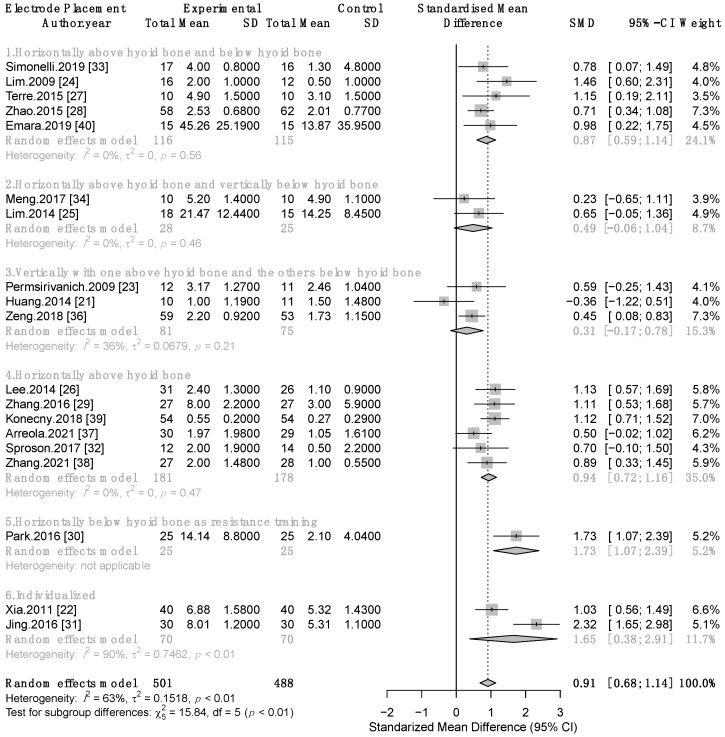
Meta-analysis forest plot of TNMES combined with CSTs versus CSTs alone on swallowing function. SD, standard deviation; CI, confidence interval; SMD, standardized mean difference.

Nineteen studies contained available data to conduct a meta-analysis comparing the effectiveness of TNMES combined with CSTs versus CSTs alone on swallowing function involving 989 patients with post-stroke dysphagia [21,22,23,24,25,26,27,28,29,30,31,32,33,34,36,37,38,39,40]. Meta-analysis revealed that the combined therapy of TNMES and CSTs significantly surpassed CSTs alone in improving swallowing function, a large effect size with moderate heterogeneity was observed (standardized mean difference (SMD) = 0.91, 95% confidence interval (95% CI): 0.68 to 1.14, *p* < 0.0001; I^2^ = 63%; test of heterogeneity *p* = 0.0001). As a result, we conducted subgroup analyses based on the varying electrode placements. Six subgroups were examined based on six distinct electrode placement procedures. The largest pooled effect size was found in two trials [22,31] that individualized electrode placement for each patient based on dysphagia assessment results (SMD = 1.65, 95 % CI: 0.38 to 2.91; I^2^ = 90%). Significant pooled effect sizes with no heterogeneity were found in two subgroups that performed Placement 1 [24,27,28,33,40] and Placement 4 [26,29,32,37,38,39] with SMD = 0.87, 95% CI: 0.59 to 1.14; I^2^ = 0% and SMD = 0.94, 95% CI: 0.72 to 1.16; I^2^ = 0%, respectively. Two subgroups applying Placement 2 [25,34] and Placement 3 [21,23,36] revealed nonsignificant pooled effect sizes with SMD = 0.49, 95% CI: −0.06 to 1.04; I^2^ = 0% and SMD = 0.31, 95% CI: −0.17 to 0.78; I^2^ = 36%, respectively. Significant effect size was observed in the study that stimulated infrahyoid as resistance training [30] (SMD = 1.73, 95% CI: 1.07 to 2.39). Test for subgroup differences *p* < 0.01 (Figure 3). 

To evaluate whether the risk of bias of the included studies influenced the results, we conducted sensitivity analyses including only studies with good quality (PEDro score ≥ 6), the results remained unchanged in this analysis (Figure S1). We then further excluded subgroups containing only one, two, and three studies in turn; the results of sensitivity analyses were consistent with the primary analysis that TNMES with Placement 1 and Placement 4 yielded significant pooled effect sizes with no heterogeneity (Figures S2 and S3).

**Figure 4:**
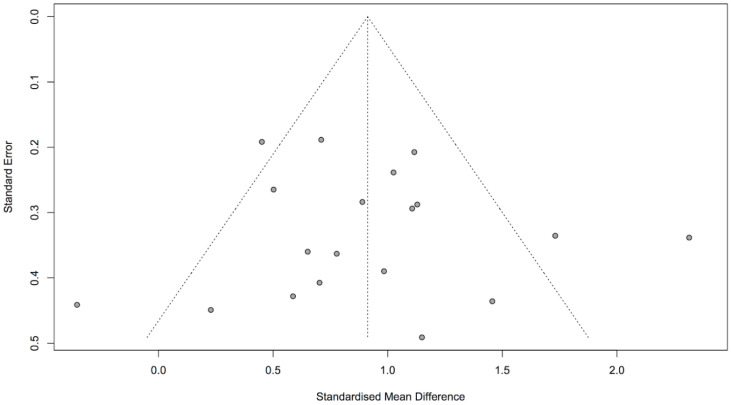
Funnel plot of TNMES combined with CSTs versus CSTs alone on swallowing function. Dots represent individual studies. Visual inspection of the funnel plot showed a symmetrical scatter of studies around the summary effect.

A funnel plot is shown in Figure 4. Publication bias was not significant (*p* = 0.7109) according to the regression test results for funnel plot asymmetry (Egger’s test).

#### 3.3.2. Therapeutic Effect of TNMES Alone Compared to CSTs on Swallowing Function

**Figure 5:**
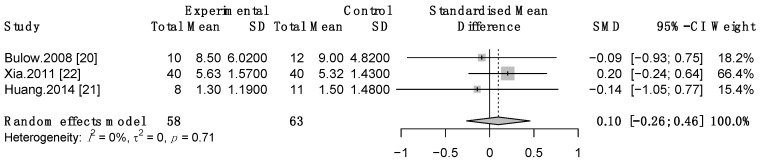
Forest plot of TNMES versus CSTs on swallowing function. SD, standard deviation; CI, confidence interval; SMD, standardized mean difference.

Three studies [20,21,22] investigated the effect of TNMES alone versus CSTs on post-stroke patients’ swallowing function. With SMD = 0.1, 95% CI: −0.26 to 0.46, *p* = 0.6; I^2^ = 0%, these studies consistently indicated that administration of TNMES alone, compared with CSTs, resulted in nonsignificant improvement in swallowing function (Figure 5). 

#### 3.3.3. Therapeutic Effect of TNMES Combined CSTs on Patients’ Quality of Life

**Figure 6:**
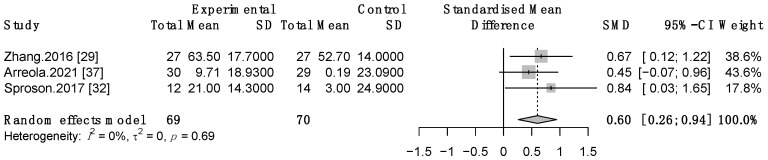
Forest plot of TNMES combined with CSTs versus CSTs alone on quality of life. SD, standard deviation; CI, confidence interval; SMD, standardized mean difference.

Three studies [29,32,37] examined the quality of life of patients who received both TNMES and CSTs compared to those who only received CSTs. Meta-analysis yielded a moderate significant effect size with no heterogeneity (SMD = 0.6, 95% CI: 0.26 to 0.94, *p* = 0.0006; I^2^ = 0%) (Figure 6).

## 4. Discussion

To the best of our knowledge, this is the first systematic review and meta-analysis to comprehensively analyze the effectiveness and the optimal electrode placement of TNMES on patients with post-stroke dysphagia as well as determine the cause of heterogeneity among trials. The findings of this study support the administration of TNMES (Placement 1 or Placement 4) in combination with CSTs in patients with post-stroke dysphagia. Moreover, it is promising to determine the dysfunctional muscles and place the electrodes accordingly in order to achieve maximal TNMES effectiveness.

The mechanisms of action of TNMES have not yet been completely elucidated. It is postulated that through eliciting muscle contractions, TNMES enhances the hyolaryngeal elevation, restores the motor function of dysfunctional muscles, and protects the dysfunctional muscles from atrophy, when applied appropriately [29]. Placement 1 and Placement 4 both include the horizontal pair of electrodes in the submental region, which stimulates suprahyoid muscles, including mylohyoid, anterior digastric, and geniohyoid muscles, pulling the hyoid forward and upward, facilitating the mechanism of airway protection and the opening of the upper esophageal sphincter [44]. In addition to one horizontal pair of electrodes above the hyoid bone, Placement 1 includes an additional horizontal pair of electrodes at the level of the thyroid notch. This channel might benefit the thyrohyoid muscle, which is thought to play a role in approximating the larynx and hyoid [45]. Notwithstanding that, supplementing one channel of electrodes to target thyrohyoid muscles in Placement 1 was not superior to Placement 4 in improving swallowing function. Further studies might be needed to compare these two regimens directly.

The vertical placement of one pair of electrodes along the midline of the neck just at and below the thyroid notch possibly induces contraction to the sternohyoid muscle rather than the thyrohyoid muscle since the sternohyoid muscle covers this muscle. There was a lack of evidence on the utilization of Placement 2 and Placement 3 in patients with post-stroke dysphagia. Moreover, in the study conducted by Huh et al., 2020, Placement 1, Placement 2, and Placement 3 were directly compared with each other. The results of this comparison fortify the findings of the present study that Placement 1 was more effective than the other two placements [41].

Remarkably, a study conducted by Park et al., 2016, administered horizontal placement of two pairs of electrodes on the region below the hyoid bone targeting infrahyoid muscles such as omohyoid, sternohyoid, and sternothyroid muscles. Patients who could swallow against the resistance produced by TNMES were recruited. During the stimulation, they were instructed to swallow forcefully with saliva or a small amount of water in order to raise the hyoid bone. This TNMES procedure revealed a significant improvement in swallowing function compared to the sham-TNMES group, suggesting that TNMES could be administered as resistance training in conjunction with effortful swallowing [30]. This procedure of infrahyoid TNMES acting as resistance was subsequently compared with suprahyoid TNMES acting as assistance; suprahyoid TNMES resulted in a significantly better result in the penetration–aspiration scale [42]. Future studies with larger sample sizes are required to confirm the efficacy of infrahyoid TNMES combined with effortful swallowing and whether this approach is superior to the other placements that target suprahyoid muscles.

Promising results were found when individualizing electrode placement according to the results of dysphagia examination. However, given the high heterogeneity among these two studies, the pooled effect size should therefore be interpreted with caution. In one study, the placements were selected based on VFSS evaluation without any further explanation on how these were arranged [22]. The other study proposed that in patients with heavy food residue and laryngeal movement defects, one pair of electrodes was horizontally attached closely at the surface of the hyoid bone, and the other pair was horizontally attached just below the superior thyroid notch; in patients with both pharyngeal and laryngeal movement dysfunctions, two pairs of electrodes were vertically placed on each side of the mid-line, and the bottom electrodes were pasted just above the superior thyroid notch; in addition to the channel placed superior to the hyoid, one channel was also distributed along the buccal branch of the seventh cranial nerve in patients with oral phase dysfunction [31]. In the study by Lee et al., 2019, patients with subacute stroke who had oral phase impairment were recruited and randomized into two groups. In one group, TNMES was administered to suprahyoid muscle, while in the other group, TNMES was applied to both suprahyoid muscle and masseter muscle simultaneously. Both groups improved their total functional dysphagia scale (FDS) scores after two weeks of treatment. In addition, the group receiving additional TNMES for masseter muscle revealed significant improvement in oral dysfunction, whereas the group that received only TNMES for the suprahyoid muscle did not [43]. Further studies should be conducted to elucidate the efficiency of electrode placement on the bilateral cheeks in patients with oral-phase dysphagia. Although instrumental assessments are not always available or prerequisite, comprehensive and objective examination should ideally be conducted to identify and prioritize specific dysfunctional muscles treated with TNMES. Further research will need to establish the specific protocol for electrode placement based on swallowing examination.

A comprehensive search strategy is one of the strengths of our systematic review. This is the first meta-analysis of the effect of TNMES on post-stroke dysphagia, resolving heterogeneity among trials, offering firm evidence regarding the therapeutic effect and the optimal electrode placement on patients with dysphagia after stroke. We acknowledge possible limitations of this systemic review and meta-analysis. Several conference’s abstracts or proceedings did not have full text available that could not be included in this systemic review; besides, we included the studies published in English only, yet the elaborate search yielded no publication bias that could be considered a strength of this study. Given the limited related evidence, it is not possible to determine what influence the time since stroke and duration of the intervention might have had on the improvement in swallowing function. In addition, some subgroups might include too few studies to obtain sufficient statistical power, which may have led to low precision in these subgroup analyses.

## 5. Conclusions

The findings of this systematic review and meta-analysis verified the therapeutic effect of combined therapy of TNMES and CSTs on swallowing function and quality of life of dysphagic patients after stroke. The management of post-stroke dysphagia with TNMES is not suggested in isolation from other rehabilitative strategies. Horizontal electrode placement should target suprahyoid muscles or simultaneously suprahyoid and thyrohyoid muscles, ideally individualized based on comprehensive dysphagia evaluation results.

## Figures and Tables

**Table 1:** Characteristics of the included studies.

No	Author, Year	Country	Sample Size	Time Since Stroke	Interventional and Control Groups (TNMES Protocol)	Electrode Placements	Outcomes and Results
1	Bulow, 2008 [20]	France, The Netherlands, Sweden	25 (12/13)	>3 months	Intervention: TNMES PD (pulse duration): 700 μs, F (frequency): 80 Hz, I (intensity): 4.5–25 mA (mean: 13 mA); 60 min/day, 5 days/week for 3 weeks Control: CSTs	Placement 1	OMFT − VFSS −
2	Permsirivanich, 2009 [23]	Thailand	23 (12/11)	>2 weeks	Intervention: TNMES + CSTs PD: 700 μs, F: 80 Hz; 60 min/day, 5 days/week for 4 weeks Control: CSTs	Placement 3	FOIS +
3	Lim, 2009 [24]	Korea	28 (16/12)		Intervention: TNMES + CSTs TNMES: PD: 700 μs, F: 80 Hz, I: approximately 7 mA; 60 min/day, 5 days/week for 4 weeks Control: CSTs	Placement 1	VFSS +
4	Xia, 2011 [22]	China	120 (40/40/40)	Subacute	Intervention 1 (I1): TNMES + CSTs Intervention 2 (I2): TNMES TNMES: PD: 700 μs, F: 80 Hz, I: 0–25 mA; 30 min sessions twice a day, 5 days/week for 4 weeks Control (C): CSTs	Electrodes were individualized according to VFSS scores, tolerance and condition of patients	SSA + VFSS + SWALQOL + I2 vs. C −
5	Lim, 2014 [25]	Korea	33 (18/15)	<3 months	Intervention: TNMES (2 weeks) + CSTs (4 weeks) TNMES: PD: 300 μs, F:80 Hz, I: 7–9 mA; 30 min/day, 5 days/week for 2 weeks Control: CSTs for 4 weeks	Placement 2	VFSS +
6	Huang, 2014 [21]	Taiwan	29 (10/11/8)	<3 months	Intervention 1 (I1): TNMES + CSTs Intervention 2 (I2): TNMES TNMES: PD: 700 μs, F: 80 Hz, I: 0–25 mA; 60 min/day, 3 days/week, totally 10 sessions Control: CSTs	Placement 3	FOIS − VFSS −
7	Lee, 2014 [26]	Korea	57 (31/26)	Within 10 days	Intervention: TNMES + CSTs TNMES: PD: 700 μs, F: 80 Hz; 30 min/day, 5 days/week + CSTs: 60 min/day, 5 days/week for 3 weeks Control: CSTs	Placement 4	FOIS +
8	Terre, 2015 [27]	Spain	20 (10/10)	1–6 months	Intervention: TNMES + CSTs TNMES: PD: 300 μs, F: 80 Hz, I: 7–19.4 mA, 60 min/day, 5 days per week for 4 weeks Control: Sham TNMES + CSTs	Placement 1	FOIS +
9	Zhao, 2015 [28]	China	120 (58/62)		Intervention: TNMES + CSTs TNMES: F: 50–100 Hz, 30 min, twice a day for 2 weeks Control: CSTs	Placement 1	WST +
10	Zhang, 2016 [29]	China	54 (27/27)	<1 month	Intervention: TNMES + CSTs TNMES: PD: 100 μs, F: 120 Hz, I: 2–60 mA, 20 min/session, twice a day, 5 days/week for 4 weeks Control: CSTs	Placement 4	SSA + WST + FOIS + SWALQOL +
11	Park, 2016 [30]	Korea	50 (25/25)	>6 months	Intervention: TNMES + CSTs TNMES: PD: 700 μs, F: 80 Hz, I: 9–14 mA, 30 min/day, 5 days/week for 6 weeks Patients performed effortful swallow to elevate the hyoid during stimulation Control: Sham TNMES + CSTs	The electrodes were located in the infrahyoid region to target the sternohyoid, omohyoid, and sternothyroid muscles	VFSS +
12	Jing, 2016 [31]	China	60 (30/30)	Within 1–3 days	Intervention: TNMES + CSTs TNMES: PD: 700 μs, F: 80 Hz, I: 6–21 mA, in 10 consecutive days Control: CSTs	Electrodes was individualized according to the result of dysphagia evaluation	SFS +
13	Sproson, 2017 [32]	The UK	26 (12/14)	>1 month	Intervention: TNMES + CSTs TNMES: F: 30 Hz, 30 min/day, 5 days/week for 4 weeks Control: CSTs	Placement 4	FOIS − VFSS − SWALQOL −
14	Simonelli, 2019 [33]	Italy	33 (17/16)	<3 months	Intervention: TNMES + CSTs TNMES: PD: 300 μs, F: 80 Hz, I: 7.8–12.5 mA, 30 min/day, 5 days/week for 8 weeks Control: CSTs	Placement 1	FOIS +
15	Meng, 2017 [34]	China	20 (10/10)	<6 months	Intervention: TNMES + CSTs TNMES: F: 80 Hz, I: 0–25 mA, 30 min/day, 5 days/week for 2 weeks Control: CSTs	Placement 2	VFSS + WST +
16	Guillen, 2017 [35]	Spain	41 (20/21)	Within 1–3 weeks	Intervention: TNMES + CSTs TNMES: F: 80 Hz, 40 min/day, 5 days/week for 3 weeks Control: CSTs	Placement 4	V-VST +
17	Zeng, 2018 [36]	China	112 (59/53)		Intervention: TNMES + CSTs TNMES: PD: 800 μs, I: 28 mA, 20 min session, once-daily for 12 days followed by a 2-day break, then continue another 12-day course of treatment Control: CSTs	Placement 3	WST +
18	Arreola, 2021 [37]	Spain	59 (30/29)	>3 months	Intervention: TNMES + CSTs TNMES: PD: 700 μs, F: 80 Hz, I: 11.86 ± 5.11 mA, 1 h session twice a day for the first week and once a day for the second week (5 days/week) Control: CSTs	Placement 4	VFSS +
19	Zhang, 2021 [38]	China	55 (27/28)	1–3 months	Intervention: TNMES + CSTs TNMES: PD: 300 μs, F: 80 Hz, I: 6.3–13.2 mA, 30 min/day, 5 days/week for 6 weeks Control: CSTs	Placement 4	VFSS +
20	Konecny, 2018 [39]	Czech Republic	108 (54/54)	Early stage after stroke	Intervention: TNMES + CSTs TNMES: PD: 300 μs, F: 60 Hz, 20 min/day, 5 days/week for 4 weeks Control: CSTs	Placement 4	VFSS +
21	Emara, 2019 [40]	Egypt	30 (15/15)	1–3 months	Intervention: TNMES + CSTs TNMES: PD: 300 μs, F: 80 Hz, I: 2.5–25 mA, 30 min/day, 3 days/week for 3 weeks Control: Sham TNMES + CSTs	Placement 1	MASA +
22	Huh, 2020 [41]	Korea	31 (10/11/10)		Intervention: 3 groups based on electrode placement:I1: Placement 1 I2: Placement 2 I3: Placement 3 PD: 300 μs, F: 80 Hz, I: 0–25 mA, 20 min/day, 5 days/week for 4 weeks		FDS and DOSS in I1 improved significantly compared to other groups
23	Oh, 2019 [42]	Korea	26 (14/12)	<6 months	Intervention: 2 groups based on electrode placement: I1: 2 pairs of electrodes targeted suprahyoid muscles. I2: 2 pairs of electrodes targeted infrahyoid muscles. PD: 700 μs, F: 80 Hz, I: 9–14 mA, 30 min/day, 5 days/week for 4 weeks Patients performed effortful swallow to elevate the hyoid during stimulation		Significant improvement in PAS scores favoring I1
24	Lee, 2019 [43]	Korea	40 (20/20)	Subacute	Intervention: 2 groups based on electrode placement: I1: 1 pair of electrodes targeted masseter and the other pair targeted suprahyoid muscles. I2: 2 pairs of electrodes targeted suprahyoid muscles. PD: 300 μs, F: 80 Hz, I: 9–14 mA, 20 min/session, twice a day, 5 days/week for 2 weeks Both groups received CSTs		FDS and pharyngeal FDS improved in both groups. I1 improved oral FDS. No significant differences between groups

TNMES: transcutaneous neuromuscular electrical stimulation; CSTs: conventional swallowing therapies; PD: pulse duration; F: frequency; I: intensity; I1: intervention 1; I2: intervention 2; OMFT: oral motor function test; VFSS: Videofluoroscopy score; FOIS: functional oral intake scale; SSA: standardized swallowing assessment; SWALQOL: Swallowing-Related Quality of Life Questionnaire; SFS: swallow function score; V-VST: volume viscosity swallow test; DOSS: dysphagia outcome and severity scale; WST: water swallow test; MASA: Mann assessment of swallowing ability; PAS: penetration-aspiration scale; FDS: functional dysphagia scale; (+): statistically significant differences between intervention and control groups; (−): no statistically significant differences between intervention and control groups.

## Data Availability

The data that support the findings of this study are available from the corresponding author, upon reasonable request.

## Supplementary Materials

The following supporting information can be downloaded at: https://www.mdpi.com/article/10.3390/life12060875/s1, Table S1: Search strategy for the different databases; Table S2: The quality of studies based on the PEDro criteria; Table S3: PRISMA checklist; Figure S1: Sensitivity analysis including only trials with low risk of bias; Figure S2: Sensitivity analysis including only trials with low risk of bias and excluding subgroups with only one study; Figure S3: Sensitivity analysis including only trials with low risk of bias and excluding subgroups with only one, two, or three studies.

## References

[1] Feigin V.L., Stark B.A., Johnson C.O., Roth G.A., Bisignano C., Abady G.G., Abbasifard M., Abbasi-Kangevari M., Abd-Allah F., Abedi V. (2021). Global, regional, and national burden of stroke and its risk factors, 1990–2019: A systematic analysis for the Global Burden of Disease Study 2019. Lancet Neurol..

[2] Banda K.J., Chu H., Kang X.L., Liu D., Pien L.C., Jen H.J., Hsiao S.S., Chou K.R. (2022). Prevalence of dysphagia and risk of pneumonia and mortality in acute stroke patients: A meta-analysis. BMC Geriatr..

[3] Cohen D.L., Roffe C., Beavan J., Blackett B., Fairfield C.A., Hamdy S., Havard D., McFarlane M., McLauglin C., Randall M. (2016). Post-stroke dysphagia: A review and design considerations for future trials. Int. J. Stroke.

[4] Hamdy S., Aziz Q., Rothwell J.C., Power M., Singh K.D., Nicholson D.A., Tallis R.C., Thompson D.G. (1998). Recovery of swallowing after dysphagic stroke relates to functional reorganization in the intact motor cortex. Gastroenterology.

[5] Huang Y.-C., Hsu T.-W., Leong C.-P., Hsieh H.-C., Lin W.-C. (2018). Clinical Effects and Differences in Neural Function Connectivity Revealed by MRI in Subacute Hemispheric and Brainstem Infarction Patients with Dysphagia After Swallowing Therapy. Front. Neurosci..

[6] Carnaby G.D., Harenberg L. (2013). What is “usual care” in dysphagia rehabilitation: A survey of USA dysphagia practice patterns. Dysphagia.

[7] Chen Y.W., Chang K.H., Chen H.C., Liang W.M., Wang Y.H., Lin Y.N. (2016). The effects of surface neuromuscular electrical stimulation on post-stroke dysphagia: A systemic review and meta-analysis. Clin. Rehabil..

[8] Sun Y., Chen X., Qiao J., Song G., Xu Y., Zhang Y., Xu D., Gao W., Li Y., Xu C. (2020). Effects of Transcutaneous Neuromuscular Electrical Stimulation on Swallowing Disorders: A Systematic Review and Meta-Analysis. Am. J. Phys. Med. Rehabil..

[9] National Institute for Health and Care Excellence (NICE) Transcutaneous Neuromuscular Electrical Stimulation for Oropharyngeal Dysphagia in Adults. 2018 NICE International Guidance No IPG634. https://www.nice.org.uk/guidance/ipg634/chapter/1-Recommendations.

[10] Chen Y.-S. (2011). Effects of electrical stimulation on peripheral nerve regeneration. BioMedicine.

[11] Chang M.C., Park S., Cho J.Y., Lee B.J., Hwang J.M., Kim K., Park D. (2021). Comparison of three different types of exercises for selective contractions of supra- and infrahyoid muscles. Sci. Rep..

[12] Humbert I.A., Poletto C.J., Saxon K.G., Kearney P.R., Crujido L., Wright-Harp W., Payne J., Jeffries N., Sonies B.C., Ludlow C.L. (2006). The effect of surface electrical stimulation on hyolaryngeal movement in normal individuals at rest and during swallowing. J. Appl. Physiol..

[13] Paik N.J., Kim S.J., Lee H.J., Jeon J.Y., Lim J.Y., Han T.R. (2008). Movement of the hyoid bone and the epiglottis during swallowing in patients with dysphagia from different etiologies. J. Electromyogr. Kinesiol..

[14] Moher D., Shamseer L., Clarke M., Ghersi D., Liberati A., Petticrew M., Shekelle P., Stewart L.A., Group P.-P. (2015). Preferred reporting items for systematic review and meta-analysis protocols (PRISMA-P) 2015 statement. Syst. Rev..

[15] Maher C.G., Sherrington C., Herbert R.D., Moseley A.M., Elkins M. (2003). Reliability of the PEDro scale for rating quality of randomized controlled trials. Phys. Ther..

[16] Wan X., Wang W., Liu J., Tong T. (2014). Estimating the sample mean and standard deviation from the sample size, median, range and/or interquartile range. BMC Med. Res. Methodol..

[17] Cohen J. (2013). Statistical Power Analysis for the Behavioral Sciences.

[18] Higgins J.P., Thompson S.G., Deeks J.J., Altman D.G. (2003). Measuring inconsistency in meta-analyses. BMJ.

[19] Lin L., Chu H. (2018). Quantifying publication bias in meta-analysis. Biometrics.

[20] Bülow M., Speyer R., Baijens L., Woisard V., Ekberg O., Bülow M., Speyer R., Baijens L., Woisard V., Ekberg O. (2008). Neuromuscular electrical stimulation (NMES) in stroke patients with oral and pharyngeal dysfunction. Dysphagia.

[21] Huang K.-L., Liu T.-Y., Huang Y.-C., Leong C.-P., Lin W.-C., Pong Y.-P. (2014). Functional outcome in acute stroke patients with oropharyngeal Dysphagia after swallowing therapy. J. Stroke Cerebrovasc. Dis..

[22] Xia W., Zheng C., Lei Q., Tang Z., Hua Q., Zhang Y., Zhu S. (2011). Treatment of post-stroke dysphagia by vitalstim therapy coupled with conventional swallowing training. Hua Zhong Ke Ji Da Xue Xue Bao. Yi Xue Ying De Wen Ban/J. Huazhong Univ. Sci. Technology. Med. Sci..

[23] Permsirivanich W., Tipchatyotin S., Wongchai M., Leelamanit V., Setthawatcharawanich S., Sathirapanya P., Phabphal K., Juntawises U., Boonmeeprakob A. (2009). Comparing the effects of rehabilitation swallowing therapy vs. neuromuscular electrical stimulation therapy among stroke patients with persistent pharyngeal dysphagia: A randomized controlled study. Chotmaihet Thangphaet/J. Med. Assoc. Thail..

[24] Lim K.B., Lee H.J., Lim S.S., Choi Y.I. (2009). Neuromuscular electrical and thermal-tactile stimulation for dysphagia caused by stroke: A randomized controlled trial. J. Rehabil. Med..

[25] Lim K.B., Lee H.J., Yoo J., Kwon Y.G. (2014). Effect of Low-Frequency rTMS and NMES on Subacute Unilateral Hemispheric Stroke With Dysphagia. Ann. Rehabil. Med..

[26] Lee K.W., Kim S.B., Lee J.H., Lee S.J., Ri J.W., Park J.G. (2014). The effect of early neuromuscular electrical stimulation therapy in acute/subacute ischemic stroke patients with Dysphagia. Ann. Rehabil. Med..

[27] Terre R., Mearin F. (2015). A randomized controlled study of neuromuscular electrical stimulation in oropharyngeal dysphagia secondary to acquired brain injury. Eur. J. Neurol..

[28] Zhao J.W., Wang Z.Y., Cao W.Z., Zhang Y.W., Song S.C., Kang W.G., Yang J.H. (2015). Therapeutic efficacy of swallowing neuromuscular electrical stimulation combined with acupuncture for post-stroke dysphagia. World J. Acupunct. Moxibustion.

[29] Zhang M., Tao T., Zhang Z.-B., Zhu X., Fan W.-G., Pu L.-J., Chu L., Yue S.-W. (2016). Effectiveness of Neuromuscular Electrical Stimulation on Patients With Dysphagia With Medullary Infarction. Arch. Phys. Med. Rehabil..

[30] Park J.S., Oh D.H., Hwang N.K., Lee J.H. (2016). Effects of neuromuscular electrical stimulation combined with effortful swallowing on post-stroke oropharyngeal dysphagia: A randomised controlled trial. J. Oral Rehabil..

[31] Jing Q., Yang X., Reng Q. (2016). Effect of neuromuscular electrical stimulation in patients with post-stroke dysphagia. Med. Sci. Technol..

[32] Sproson L., Pownall S., Enderby P., Freeman J. (2018). Combined electrical stimulation and exercise for swallow rehabilitation post-stroke: A pilot randomized control trial. Int. J. Lang. Commun. Disord..

[33] Simonelli M., Ruoppolo G., Iosa M., Morone G., Fusco A., Grasso M.G., Gallo A., Paolucci S. (2019). A stimulus for eating. The use of neuromuscular transcutaneous electrical stimulation in patients affected by severe dysphagia after subacute stroke: A pilot randomized controlled trial. NeuroRehabilitation.

[34] Meng P., Zhang S., Wang Q., Wang P., Han C., Gao J., Yue S. (2018). The effect of surface neuromuscular electrical stimulation on patients with post-stroke dysphagia. J. Back Musculoskelet. Rehabil..

[35] Guillén-Solà A., Messagi Sartor M., Bofill Soler N., Duarte E., Barrera M.C., Marco E. (2017). Respiratory muscle strength training and neuromuscular electrical stimulation in subacute dysphagic stroke patients: A randomized controlled trial. Clin. Rehabil..

[36] Zeng Y., Yip J., Cui H., Guan L., Zhu H., Zhang W., Du H., Geng X. (2018). Efficacy of neuromuscular electrical stimulation in improving the negative psychological state in patients with cerebral infarction and dysphagia. Neurol. Res..

[37] Arreola V., Ortega O., Alvarez-Berdugo D., Rofes L., Tomsen N., Cabib C., Muriana D., Palomera E., Clave P. (2021). Effect of Transcutaneous Electrical Stimulation in Chronic Poststroke Patients with Oropharyngeal Dysphagia: 1-Year Results of a Randomized Controlled Trial. Neurorehabilit. Neural Repair.

[38] Zhang Q., Wu S. (2021). Effects of Synchronized Neuromuscular Electrical Stimulation (NMES) on the Submental Muscles During Ingestion of a Specified Volume of Soft Food in Patients with Mild-to-Moderate Dysphagia Following Stroke. Med. Sci. Monit..

[39] Konecny P., Elfmark M., Rosolova M., Bastlova P., Lerchova I., Muckova A. (2017). Electrical Stimulation of the Suprahyoid Muscles in Post Stroke Patients with Dysphagia. Ceska a Slovenska Neurologie a Neurochirurgie.

[40] Emara T. (2019). Effect of transcutaneous electrical nerve stimulation and conventional therapy in post-stroke dysphagic patients: A randomized controlled trial. Biosci. Res..

[41] Huh J.W., Park E., Min Y.S., Kim A.R., Yang W.J., Oh H.M., Nam T.W., Jung T.D. (2020). Optimal placement of electrodes for treatment of post-stroke dysphagia by neuromuscular electrical stimulation combined with effortful swallowing. Singap. Med. J..

[42] Oh D.-H., Park J.-S., Kim H.-J., Chang M.-Y., Hwang N.-K. (2020). The effect of neuromuscular electrical stimulation with different electrode positions on swallowing in stroke patients with oropharyngeal dysphagia: A randomized trial. J. Back Musculoskelet. Rehabil..

[43] Lee K.W., Kim S.B., Lee J.H., Lee S.J., Park J.G., Jang K.W. (2019). Effects of Neuromuscular Electrical Stimulation for Masseter Muscle on Oral Dysfunction After Stroke. Ann. Rehabil. Med..

[44] Rofes L., Arreola V., López I., Martin A., Sebastián M., Ciurana A., Clavé P. (2013). Effect of surface sensory and motor electrical stimulation on chronic poststroke oropharyngeal dysfunction. Neurogastroenterol. Motil..

[45] Pearson W.G., Langmore S.E., Yu L.B., Zumwalt A.C. (2012). Structural analysis of muscles elevating the hyolaryngeal complex. Dysphagia.

