# Supplementary material for: Therapeutic Effect and Optimal Electrode Placement of Transcutaneous Neuromuscular Electrical Stimulation in Patients with Post-Stroke Dysphagia: A Systematic Review and Meta-Analysis of Randomized Controlled Trials
Source: Life (Basel). 2022 Jun 10;12(6):875. doi: 10.3390/life12060875 (PMC9225155; doi:10.3390/life12060875)
Supplement: Supplementary file 1 [file life-12-00875-s001.zip › life-1738161-supplementary.pdf]

Supplementary material for the paper:

Therapeutic Effect and Optimal Electrode Placement of Transcutaneous Neuromuscular Electrical Stimulation in Patients with Post-Stroke Dysphagia: A Systematic Review and Meta-analysis of Randomized Controlled Trials

Table S1: Search Strategy for the different databases

Database	Search Strategy
**EMBASE**	(dysphagia:ab,ti OR swallow*:ab,ti OR deglutition:ab,ti OR oropharyn*:ab,ti) AND (electrotherapy:ab,ti OR electric*:ab,ti OR stimulat*:ab,ti OR electrotherap*:ab,ti OR vitalstim:ab,ti OR suprahyoid:ab,ti OR infrahyoid:ab,ti OR neurostim:ab,ti) AND ('cerebrovascular accident':ab,ti OR 'cerebral ischemi*':ab,ti OR cerebrovascular:ab,ti OR 'cerebral embolism':ab,ti OR cerebrovascular*:ab,ti OR intracranial:ab,ti OR hemiparesis:ab,ti OR hemiplegia:ab,ti OR medulla*:ab,ti OR tia:ab,ti) AND [humans]/lim
**CINAHL**	AB (dysphagia or swallowing disorders or deglutition disorders or swallowing or swallow or oropharyn*) AND AB (electrical stimulation or functional electrical stimulation or neuromuscular electrical stimulation or fes or nmes or stimulat* or electric* or electrotherap*) AND AB (stroke or cerebrovascular accident or cva or cerebral vascular event or cve or transient ischaemic attack or tia or medulla*)
**Web of Science**	((AB=(swallow* or dysphag* or deglutit* or oropharyn*)) AND AB=(electrotherapy or electric* or stimulat* or electrotherap* or neurostim* or vitalstim or neurostim or NMES or TES or infrahyoid or suprahyoid)) AND AB=(stroke or cerebrovascular* or cerebral ischemi* or CVA or intracranial or cerebral embolism or hemiparesis or hemiplegia or medulla*)
**Pubmed**	((stroke) OR (transient ischemic attack*) OR (TIA) OR (brain hypoxia) OR (brain infarct*) OR (cerebral haemorrhage) OR (cerebral ischemia) OR (cerebral ischemi*) OR (cerebrovascular disease) OR (cerebrovascular stroke) OR (cerebral embolism) OR (brain haemorrhage) OR (brain infarct*) OR (cerebrovascular accident*) OR (CVA) OR (cerebrovascular*) OR (cerebrovascular disorder) OR (Ischemic stroke) OR (Ischemic apoplexy) OR (intracranial embolism) OR (Intracranial thrombosis) OR (hemiparesis) OR (hemiplegia)) AND ((*swallow*) OR (*dysphag*) OR (deglutit*) OR (*pharyn*) OR (Deglutition [Mesh]) OR (dysphagia [Mesh])) AND ((*electr*)

	OR (electrical stimulation) OR (NMES) OR (vitalstim) OR (*stimul*) OR (Electric Stimulation Therapy [Mesh]) OR (Electric Stimulation [Mesh]))<br>Article Type: Randomized Controlled Trial
**Cochrane**	**#1** **MeSH descriptor: [Deglutition Disorders] explode all trees**<br>**#2** MeSH descriptor: [Deglutition] explode all trees<br>**#3** swallow* or deglutit* or dysphag*<br>**#4** Oropharyngeal<br>**#5** MeSH descriptor: [Electric Stimulation] explode all trees<br>**#6** electric* or electro*<br>**#7** stimulat*<br>**#8** electrotherap*<br>**#9** NMES OR VitalStim OR Suprahyoid OR infrahyoid<br>**#10** MeSH descriptor: [Stroke] explode all trees<br>**#11** MeSH descriptor: [Cerebrovascular Disorders] explode all trees<br>**#12** MeSH descriptor: [Hemiplegia] explode all trees<br>**#13** medulla*<br>**#14** Poststroke OR Post-stroke<br>**#15** #1 OR #2 OR #3 OR #4<br>**#16** #5 OR #6 OR #7 OR #8 OR #9<br>**#17** #10 OR #11 OR #12 OR #13 OR #14<br>**#18** #15 AND #16 AND #17<br>Limits in trials

Table S2: The quality of studies basing on the PEDro criteria

Author, year	Specified eligibility criteria	Random allocation	Concealment of allocation	Baseline comparability	Blinding of subjects	Blinding of therapist	Blinding of assessors	Adequate follow-up	Intention-to-treat analysis	Between-group comparisons	Point estimate and variability	Total PEDro Score
Bulow, 2008	1	1	0	1	0	0	0	0	1	1	1	5
Permsirivanich, 2009	1	1	1	1	0	0	0	0	1	1	1	6
Lim, 2009	1	1	0	1	0	0	1	0	0	1	1	5
Xia, 2011	1	1	1	1	0	0	1	1	1	1	1	8
Lim, 2014	1	1	1	1	0	0	0	0	1	1	1	6
Huang, 2014	1	1	0	1	0	0	1	1	0	1	1	6
Lee, 2014	1	1	0	1	0	0	0	1	0	1	1	5
Terre, 2015	1	1	1	1	1	0	1	0	0	1	1	7
Zhao, 2015	1	1	1	1	0	0	0	1	1	1	1	7
Zhang, 2016	1	1	1	1	0	0	0	1	0	1	1	6
Park, 2016	1	1	0	1	0	0	1	1	0	1	1	6
Jing, 2016	1	1	1	1	0	0	1	1	1	1	1	8
Sproson,2017	1	1	1	1	0	0	1	1	1	1	1	8
Simonelli, 2019	1	1	1	1	0	0	1	1	0	1	1	7
Meng, 2017	1	1	0	1	0	0	0	1	0	1	1	5
Guillen, 2017	1	1	1	1	0	0	1	0	1	1	1	7
Zeng, 2018	1	1	1	1	0	0	1	1	1	1	1	8
Arreola, 2021	1	1	1	1	0	0	1	1	0	1	1	7
Zhang, 2021	1	1	1	1	0	0	1	1	1	1	1	8
Konecny, 2018	1	1	1	1	0	0	0	1	1	1	1	7
Emara, 2019	1	1	1	1	1	0	0	1	0	1	1	7
Huh, 2020	1	1	1	1	0	0	1	1	1	1	1	8
Oh, 2019	1	1	1	1	0	0	1	0	1	1	1	7
Lee, 2019	1	1	0	1	0	0	0	1	0	1	1	5

Table S3: PRISMA Checklist

Section/topic	#	Checklist item	Reported on page #
**TITLE**			
Title	1	Identify the report as a systematic review, meta-analysis, or both.	1
**ABSTRACT**			
Structured summary	2	Provide a structured summary including, as applicable: background; objectives; data sources; study eligibility criteria, participants, and interventions; study appraisal and synthesis methods; results; limitations; conclusions and implications of key findings; systematic review registration number.	1
**INTRODUCTION**			
Rationale	3	Describe the rationale for the review in the context of what is already known.	2
Objectives	4	Provide an explicit statement of questions being addressed with reference to participants, interventions, comparisons, outcomes, and study design (PICOS).	2
**METHODS**			
Protocol and registration	5	Indicate if a review protocol exists, if and where it can be accessed (e.g., Web address), and, if available, provide registration information including registration number.	2
Eligibility criteria	6	Specify study characteristics (e.g., PICOS, length of follow-up) and report characteristics (e.g., years considered, language, publication status) used as criteria for eligibility, giving rationale.	2-3
Information sources	7	Describe all information sources (e.g., databases with dates of coverage, contact with study authors to identify additional studies) in the search and date last searched.	3
Search	8	Present full electronic search strategy for at least one database, including any limits used, such that it could be repeated.	3, supplement
Study selection	9	State the process for selecting studies (i.e., screening, eligibility, included in systematic review, and, if applicable, included in the meta-analysis).	2,3
Data collection process	10	Describe method of data extraction from reports (e.g., piloted forms, independently, in duplicate) and any processes for obtaining and confirming data from investigators.	3
Data items	11	List and define all variables for which data were sought (e.g., PICOS, funding sources) and any assumptions and simplifications made.	3
Risk of bias in individual studies	12	Describe methods used for assessing risk of bias of individual studies (including specification of whether this was done at the study or outcome level), and how this information is to be used in any data synthesis.	3, supplement
Summary measures	13	State the principal summary measures (e.g., risk ratio, difference in means).	3
Synthesis of results	14	Describe the methods of handling data and combining results of studies, if done, including measures of consistency (e.g., I^2) for each meta-analysis.	3,4

Section/topic	#	Checklist item	Reported on page #
Risk of bias across studies	15	Specify any assessment of risk of bias that may affect the cumulative evidence (e.g., publication bias, selective reporting within studies).	3,4
Additional analyses	16	Describe methods of additional analyses (e.g., sensitivity or subgroup analyses, meta-regression), if done, indicating which were pre-specified.	3
**RESULTS**			
Study selection	17	Give numbers of studies screened, assessed for eligibility, and included in the review, with reasons for exclusions at each stage, ideally with a flow diagram.	4
Study characteristics	18	For each study, present characteristics for which data were extracted (e.g., study size, PICOS, follow-up period) and provide the citations.	4,5
Risk of bias within studies	19	Present data on risk of bias of each study and, if available, any outcome level assessment (see item 12).	4, Supplement
Results of individual studies	20	For all outcomes considered (benefits or harms), present, for each study: (a) simple summary data for each intervention group (b) effect estimates and confidence intervals, ideally with a forest plot.	6-12
Synthesis of results	21	Present results of each meta-analysis done, including confidence intervals and measures of consistency.	10-12
Risk of bias across studies	22	Present results of any assessment of risk of bias across studies (see Item 15).	11
Additional analysis	23	Give results of additional analyses, if done (e.g., sensitivity or subgroup analyses, meta-regression [see Item 16]).	10
**DISCUSSION**			
Summary of evidence	24	Summarize the main findings including the strength of evidence for each main outcome; consider their relevance to key groups (e.g., healthcare providers, users, and policy makers).	12,13
Limitations	25	Discuss limitations at study and outcome level (e.g., risk of bias), and at review-level (e.g., incomplete retrieval of identified research, reporting bias).	13
Conclusions	26	Provide a general interpretation of the results in the context of other evidence, and implications for future research.	13
**FUNDING**			
Funding	27	Describe sources of funding for the systematic review and other support (e.g., supply of data); role of funders for the systematic review.	14

Electrode Placement Author.year	Experimental Total	Experimental Mean	Experimental SD	Control Total	Control Mean	Control SD	SMD	95%−CI	Weight
**1.Horizontally above hyoid bone and below hyoid bone**									
Simonelli.2019	17	4.00	0.8000	16	1.30	4.8000	0.78	[0.07; 1.49]	5.6%
Terre.2015	10	4.90	1.5000	10	3.10	1.5000	1.15	[0.19; 2.11]	4.1%
Zhao.2015	58	2.53	0.6800	62	2.01	0.7700	0.71	[0.34; 1.08]	8.4%
Emara.2019	15	45.26	25.1900	15	13.87	35.9500	0.98	[0.22; 1.75]	5.3%
**Random effects model**	**100**			**103**			**0.80**	**[0.51; 1.09]**	**23.4%**
Heterogeneity: $I^2 = 0\%$, $\tau^2 = 0$, $p = 0.81$									
**2.Horizontally above hyoid bone and vertically below hyoid bone**									
Lim.2014	18	21.47	12.4400	15	14.25	8.4500	0.65	[−0.05; 1.36]	5.7%
**Random effects model**	**18**			**15**			**0.65**	**[−0.05; 1.36]**	**5.7%**
Heterogeneity: not applicable									
**3.Vertically with one above hyoid bone and the others below hyoid bone**									
Permsirivanich.2009	12	3.17	1.2700	11	2.46	1.0400	0.59	[−0.25; 1.43]	4.8%
Huang.2014	10	1.00	1.1900	11	1.50	1.4800	−0.36	[−1.22; 0.51]	4.6%
Zeng.2018	59	2.20	0.9200	53	1.73	1.1500	0.45	[0.08; 0.83]	8.3%
**Random effects model**	**81**			**75**			**0.31**	**[−0.17; 0.78]**	**17.7%**
Heterogeneity: $I^2 = 36\%$, $\tau^2 = 0.0679$, $p = 0.21$									
**4.Horizontally above hyoid bone**									
Zhang.2016	27	8.00	2.2000	27	3.00	5.9000	1.11	[0.53; 1.68]	6.7%
Konecny.2018	54	0.55	0.2000	54	0.27	0.2900	1.12	[0.71; 1.52]	8.1%
Arreola.2021	30	1.97	1.9800	29	1.05	1.6100	0.50	[−0.02; 1.02]	7.1%
Sproson.2017	12	2.00	1.9000	14	0.50	2.2000	0.70	[−0.10; 1.50]	5.0%
Zhang.2021	27	2.00	1.4800	28	1.00	0.5500	0.89	[0.33; 1.45]	6.8%
**Random effects model**	**150**			**152**			**0.91**	**[0.66; 1.15]**	**33.7%**
Heterogeneity: $I^2 = 2\%$, $\tau^2 = 0.0014$, $p = 0.40$									
**5.Horizontally below hyoid bone as resistance training**									
Park.2016	25	14.14	8.8000	25	2.10	4.0400	1.73	[1.07; 2.39]	6.0%
**Random effects model**	**25**			**25**			**1.73**	**[1.07; 2.39]**	**6.0%**
Heterogeneity: not applicable									
**6.Individualized**									
Xia.2011	40	6.88	1.5800	40	5.32	1.4300	1.03	[0.56; 1.49]	7.5%
Jing.2016	30	8.01	1.2000	30	5.31	1.1000	2.32	[1.65; 2.98]	6.0%
**Random effects model**	**70**			**70**			**1.65**	**[0.38; 2.91]**	**13.5%**
Heterogeneity: $I^2 = 90\%$, $\tau^2 = 0.7462$, $p < 0.01$									
**Random effects model**	**444**			**440**			**0.90**	**[0.65; 1.16]**	**100.0%**
Heterogeneity: $I^2 = 66\%$, $\tau^2 = 0.1626$, $p < 0.01$									
Test for subgroup differences: $\chi^2_5 = 14.21$, df = 5 ($p = 0.01$)									

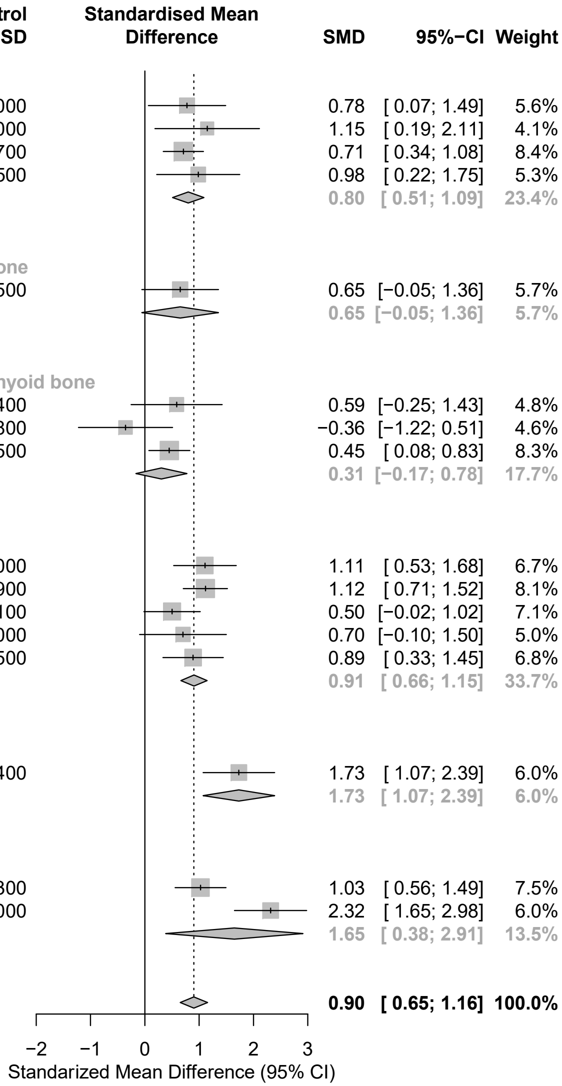

Figure S1: Sensitivity analysis including only trials with low risk of bias

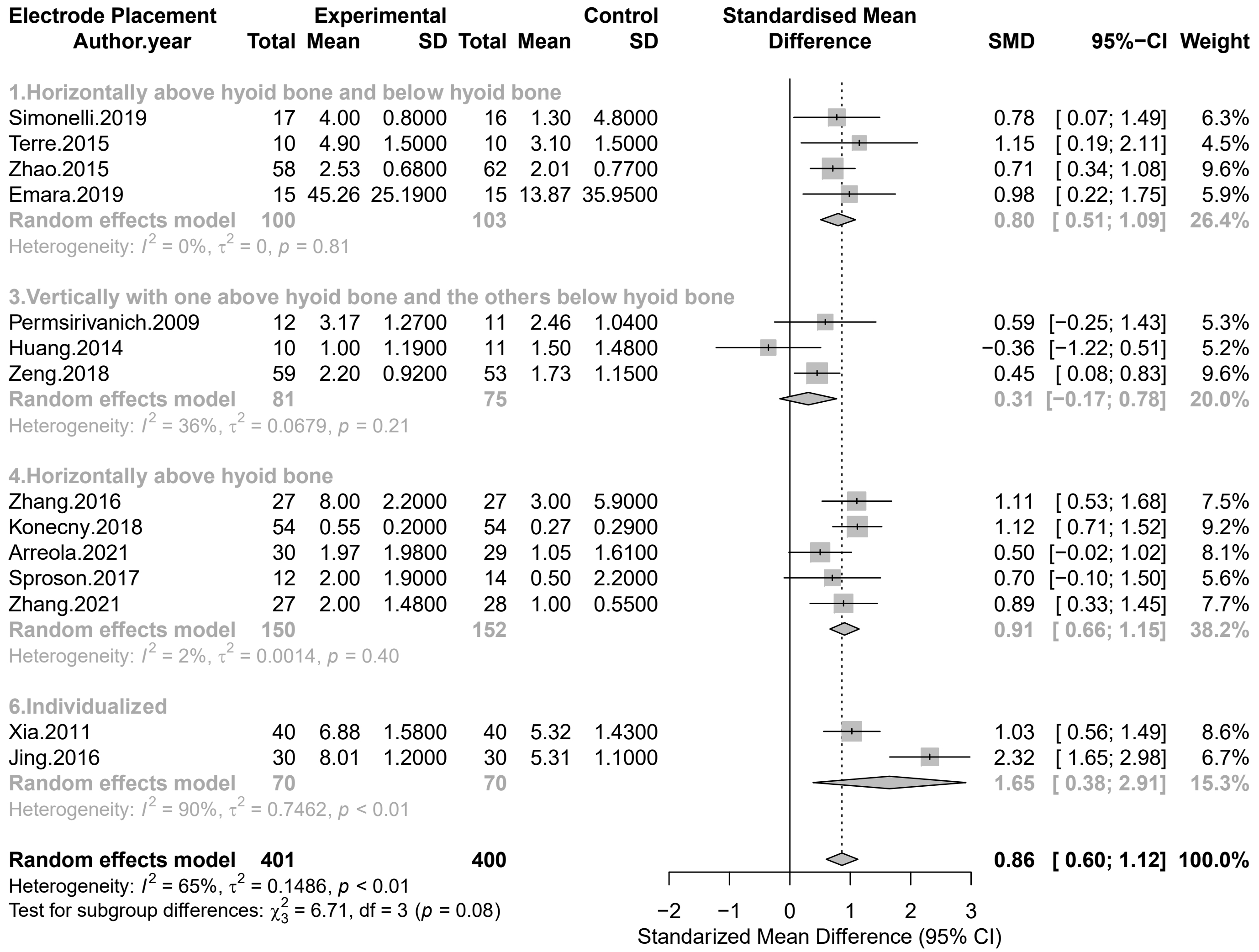

Figure S2: Sensitivity analysis including only trials with low risk of bias and excluding subgroups with only one study

Electrode Placement Author.year	Experimental Total	Mean	SD	Control Total	Mean	SD	SMD	95%−CI	Weight
**1.Horizontally above hyoid bone and below hyoid bone**									
Simonelli.2019	17	4.00	0.8000	16	1.30	4.8000	0.78	[0.07; 1.49]	6.7%
Terre.2015	10	4.90	1.5000	10	3.10	1.5000	1.15	[0.19; 2.11]	3.6%
Zhao.2015	58	2.53	0.6800	62	2.01	0.7700	0.71	[0.34; 1.08]	24.7%
Emara.2019	15	45.26	25.1900	15	13.87	35.9500	0.98	[0.22; 1.75]	5.8%
Random effects model	100			103			0.80	[0.51; 1.09]	40.8%
Heterogeneity: $I^2 = 0\%$, $\tau^2 = 0$, $p = 0.81$									
**4.Horizontally above hyoid bone**									
Zhang.2016	27	8.00	2.2000	27	3.00	5.9000	1.11	[0.53; 1.68]	10.2%
Konecny.2018	54	0.55	0.2000	54	0.27	0.2900	1.12	[0.71; 1.52]	20.4%
Arreola.2021	30	1.97	1.9800	29	1.05	1.6100	0.50	[−0.02; 1.02]	12.5%
Sproson.2017	12	2.00	1.9000	14	0.50	2.2000	0.70	[−0.10; 1.50]	5.3%
Zhang.2021	27	2.00	1.4800	28	1.00	0.5500	0.89	[0.33; 1.45]	10.9%
Random effects model	150			152			0.91	[0.66; 1.15]	59.2%
Heterogeneity: $I^2 = 2\%$, $\tau^2 = 0.0014$, $p = 0.40$									
**Random effects model**	**250**			**255**			**0.86**	**[0.68; 1.05]**	**100.0%**

Heterogeneity: $I^2 = 0\%$, $\tau^2 = 0$, $p = 0.72$

Test for subgroup differences: $\chi^2_1 = 0.31$, df = 1 ($p = 0.58$)

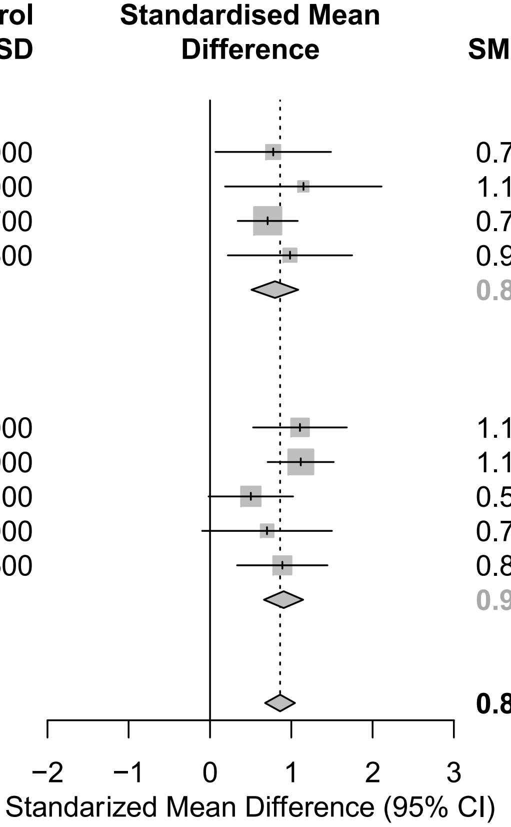

Figure S3: Sensitivity analysis including only trials with low risk of bias and excluding subgroups with only one, two or three studies
